# Positive and negative actions early in the relationship predict later interactions among toddlers

**DOI:** 10.1371/journal.pone.0276932

**Published:** 2022-11-03

**Authors:** Ayelet Lahat, Zhangjing Lou, Michal Perlman, Nina Howe, Jonathan B. Santo, Holly E. Recchia, William M. Bukowski, Hildy S. Ross

**Affiliations:** 1 OISE, University of Toronto, Toronto, Canada; 2 Concordia University, Montreal, Canada; 3 University of Nebraska, Omaha, Nebraska; 4 University of Waterloo, Waterloo, Canada; Federal University of Ceara, UNITED STATES

## Abstract

Very little is known about the role of early interactions in the development of peer relationships among toddlers. The present study examined whether behaviors early in the formation of toddler relationships predict interactions later in their relationships. Twenty-eight unfamiliar 20- and 30-month-old toddlers from a predominately European background met separately with each of two other toddlers for 18 playdates. Both positive and negative behaviors at the beginning of the relationship predicted a higher frequency of games later in the relationship. Positive behaviors at the beginning of the relationship predicted fewer conflicts later in the relationship. Negative behaviors at the beginning predicted more conflicts later in the relationship. These findings suggest that toddlers’ behaviors, when they initially meet, underlie the pathway in which their relationship develops.

## 1. Introduction

Toddler peer relationships are important for children’s socialization and set the stage for later social, emotional, and cognitive development [1, 2]. Despite the importance of young children’s friendships, less is known about how children initially form relationships with peers, particularly during toddlerhood. Initial interactions between unfamiliar peers contribute to future interactions and the development of relationships [3]. The present study examined 20- and 30-month-old unfamiliar toddlers over 18 playdates. We examined whether positive and negative behaviors early in the relationship predicted the types of interactions these toddlers engaged in later, as they got to know one another. To the best of our knowledge this is the first study to examine how initial interactions among toddlers are associated with the development of peer relationships.

Very few studies have examined how preschool-aged children’s initial interactions predict subsequent interactions. Gottman [4] examined how initial positive interactions contribute to children’s developing relationships; unfamiliar 3- to 9-year-old children were characterized as “hitting it off” when they interacted in a connected manner, exchanged information successfully, managed conflict, and established common ground. This study examined the formation of relationships over only three sessions and did not study toddlers. The present study examined the formation of relationships in a younger age group (20–30 months) over a longer period (18 sessions over 4 months).

Research with older children has documented how children’s interaction style and initial behaviors can contribute to their developing relationships. In a study with 5-year-olds observed with unfamiliar peers, those who were more aggressive during the interaction also received more aggression from their peer [5]. In a different study, second-grade boys who had aggressive tendencies, were also hostile and noncooperative with peers, thereby reducing their likelihood of forming new peer relationships [6]. In a study based on laboratory observations of dyads of unfamiliar 5^th^ graders, Andrews et al. [7] found that dyads who were more discrepant in their teacher-rated level of aggression, collaborated less and had less positive perceptions of one another. Taken together, these studies suggest that initial positive interactions between peers can enhance their developing relationship, while negative interactions can impede them.

It is likely, however, that friendship involves more complex interactions than research on first impressions may suggest. For example, relative to nonfriends, friends not only engage in more positive interactions but also demonstrate more negative interactions such as quarrelling, active hostility (threats and assaults), and reactive hostility (resistance and refusals) [8, 9]. In a study with toddlers, multiplex relationships have been observed [10], in which positive and negative behaviors appeared to be fully integrated parts of social relations among these toddlers. These findings suggest that negative behaviors may constitute normative aspects of relationships, including among toddlers.

The types of interactions toddlers engage in as they become acquainted could point to the quality of their relationship. Compared to nonfriends, friends engage in more positive physical contact and express more positive affect [11]. Friends also engage in more negative behavior, such as conflict, teasing, and competition [12]. Thus, examining change over time in positive (e.g., games) and negative (e.g., conflicts) interactions, can provide important information on toddlers’ developing relationship.

During the toddler years, young children begin to engage in coordinated and reciprocal interactions with their peers [13]. Both positive and negative overtures are given and received. For example, in a study with 2- and 3-year-old toddlers [14], previous sharing by a partner resulted in greater sharing by 3-year-olds, but not by 2-year-olds.

Using the dataset employed in the present study, Ross and Lollis [15] found reciprocal relationship effects for games sequences, while individual characteristics contributed to initiations of conflict. Thus, specific pairs interacted more positively with one another in comparison with each individual’s interaction with a partner. In contrast, more negative sequences of interaction tended to be more characteristic of individual toddlers who both displayed and elicited negative behavior from their peers. Therefore, engagement in negative interaction is associated with individual differences. The present study will extend these findings by examining how positive and negative behaviors at the beginning of the relationship contribute to toddlers’ interactions later in the relationship. In the present study 20- and 30-month-old unfamiliar toddlers were each paired with two other toddlers and met for 18 play sessions in each other’s homes (total of 36 play session for each child). Given that initial positive interactions among children promote relationship formation [4], we predicted that increased positive actions at the beginning of the relationship would be associated with a higher frequency of games sequences and lower frequency of conflicts later in the relationship. Given that initial negative perceptions of peers impede relationship formation [7], we predicted that increased negative actions at the beginning of the relationship would be associated with a higher frequency of conflicts and lower frequency of games later in the relationship.

## 2. Methods

### 2.1 Participants

The research was approved by the Office of Human Research, University of Waterloo and the Concordia University Office of Research (ethics approval number 30004857). Parents were contacted by phone based on advertisements of birth in a local newspaper. The study procedure and time commitment were explained, and oral consent was obtained for a home visit. At the initial home visit the procedure was more fully explained, questions were answered, and written consent obtained from the parents. Data were collected from 20- and 30-month-old toddlers who were from a predominantly European cultural background. Participants included 27 dyads of toddlers who were unfamiliar to one another at the beginning of the study. Each participant met with two other participants for 18 playdates for a total of 36. Same-age and same-sex toddlers were observed in dyads and arranged into 8 groups of 4 pairs. Therefore, each participant was paired with two others in a quad (i.e., AB, BC, CD, DA), with age and sex distributed equally across the quads. Age was calculated as the toddler’s age in the first session with a peer. The average age for participants in the 20-month-old quads was 21.24 months (Range 18.75 to 24.80 months); the average age for participants in the 30-month-old quads was 30.77 months (Range 28.35 to 33.95 months). The average level of parental education in the sample was 14.18 years for mothers and 16.18 years for fathers. None of the toddlers attended organized childcare. This underscores the homogeneity of our sample as none of the toddlers had previous experiences with peers in group settings, such as children who attended some form of childcare. This allows for examining the formation of new relationships, without the potential confound of other toddlers’ previous social experiences with children in a group setting. Data from one 20-month-old female quad (4 dyads), and one 30-month-old male dyad were lost since the original data collection in 1983–1984 and were not available. Therefore, the present study included data from a total of 28 toddlers formed into 27 dyads.

### 2.2 Procedure

Toddlers were observed as they played together in dyads in each other’s homes. Playdates were 40 minutes long and mothers of both children were present. Mothers were asked to allow toddlers to interact freely with one another and not to direct or organize play; they were free to respond to the toddlers’ overtures. Playdates alternated between the participants’ homes. All playdates were held within a 4-month period. The average number of days that passed from the first playdate with one peer and first playdate with the second peer was 5.15 days (SD = 5.42).

We followed observation procedures established by Dunn & Munn [16]. Five observers worked in pairs, with each pair responsible for observing all sessions within a dyad. Each session, one observer was present in the home and recorded the play session. The observer followed the children around the home, dictating all peer-related social actions onto one track of an audio-tape recorder. On a second track the children’s verbal and vocal behavior was recorded. Live observations with audio recordings were preferred because of the difficulty of following two children given that they were allowed to move freely throughout their homes. Observers later reviewed the audio tapes and coded the peer directed actions of each child. Furthermore, the coded data was accompanied by narrative descriptions of the children’s observed verbal, vocal, and nonverbal actions.

### 2.3 Coding

The research assistant observing the session transcribed and coded peer directed actions produced by each toddler. Eighty-three types of actions were coded. Of these, eleven were classified as positive and involved actions in which children express positive emotions, agreement, and sensitivity to others’ overtures. The positive actions included in the present study were making positive contact, making verbal offers, offering, giving, expressing agreement (i.e., approval using “okay”, “yes”, etc.), expressing thanks, greeting, smiling, laughing, and nodding (i.e., nod head, “yes”). Thirteen actions were classified as negative and involved actions that serve as evidence for a lack of cooperation, negative affect, threatening, and aggressive behavior. The negative actions included in the present study were causing bodily harm, expressing threat, throwing an object at the peer, pushing away or pulling, tugging, pushing an object, taking a toy, withdrawing an object, offering/withdrawing (i.e., tease, offer and then withdrawing an object), expressing disagreement, resisting, expressing protest, and fussing. Actions that were neither positive nor negative (e.g., describing/naming self or peer, touch object, vocalize, etc.) were not included here.

Actions made up interactional *sequences* between toddlers that were contiguous in time and thematically related and were coded dyadically. The present study focused specifically on games [17] and conflict sequences [18], inasmuch as both involve dyadic interactions and contribute to the development of toddler relationships [1, 15]. *Games* entailed mutually involved structured interchanges, in which toddlers have roles to play with alternating turns. Games had a playful quality with no negative affect or an apparent serious purpose [17]. For example, Child A started jumping on the couch and child B joined; both children imitated and followed each other while climbing/jumping on the couch and running around the coffee table. *Conflicts* entailed mutual opposition in individual goals and desires, and included protesting, resisting, or retaliating against the others’ actions. Conflicts included incompatibility of behavior among the peers [18]. For example, child A tugged a wooden car, and child B resisted. Child A then relinquished the car.

Five research assistants worked in various teams of two to observe during the sessions. Each pair was responsible for observing all sessions for a quad. Interobserver agreement was established by the research coordinator independently observing and coding 21 sessions. Reliability for the different codes ranged from .83 - .93 for total proportion of agreements.

### 2.4 Data analysis

In the present study, a measure of sequence frequency was derived for each type of interaction. Moves made by mothers were removed from the analysis as we were interested in peer interaction. Sequence frequency was a dyadic measure and was calculated for each playdate by summing the occurrences of each sequence type. Two dependent variables (frequency of games and conflicts) were analyzed using a 3-level cross-classified multi-level model (CCMM) to account for the nested and cross-classified structure of the data [19]. The CCMM was carried out using Stata/IC Version 16.1. In the present study, play sessions were nested within child, and children were nested within dyad. Cross-classified models were used to account for the fact that each child was a member of two different dyads (e.g., child B is in both dyad AB and dyad BC). The quad level was not included in the model as a level because of its relatively small sample size (*N* = 7). To examine the effect of membership in a quad, separate analyses were carried out, in which each quad was dummy coded and added to the model respectively as a predictor. These analyses indicate that some quads were different than others in frequency and length of various sequences. Given the small number of quads it may not be possible to draw meaningful conclusions from these results (see S1 and S2 Tables). The Akaike Information Criterion (AIC) was calculated to assess model fit; a difference of 10 in AIC value between two models is considered meaningful [20]. Frequencies of games and conflicts for each session of each toddler within each dyad (18 sessions X 2 partners X 27 dyads = 972) were inspected for outliers using boxplots. All outliers (31 games and 12 conflicts out of 972) were winsorized (i.e., replaced by the highest/smallest value in the rest of the data) [21].

When graphing each child’s average sequence frequency across the 18 play sessions for each sequence type, no clear pattern emerged (see S1 and S2 Figs). It is possible that differences among play sessions could be a result of noise in the data (e.g., a participant was tired or hungry in a particular playdate, toys available during the playdate, etc.). Therefore, the 18 play sessions were grouped into three relationship phases; the early phase included the first six play sessions, the middle phase included the middle six play sessions, and the late phase included the final six play sessions. Early, middle, and late phases were added as predictors in the CCMMs.

Hypotheses regarding change over time were tested by three CCMMs with 3 levels (i.e., session, child, dyad). The first model (i.e., the Null Model) did not include any predictors. In Model 1, the effects of phase (early, middle, late) were entered to examine the change of interactions over time. The middle and late phase were dummy coded and entered in the model to make comparisons to the early phase. The early and late phases were dummy coded and entered in as a follow-up analysis to compare the early and late phases to the middle phase. These dummy codes allow us to make all comparisons among the three different phases of the relationship. In Model 2, the main effects of positive/negative actions in the early phase and age were entered into the model. In this model we also entered as predictors the interactions between early phase positive/negative actions and phase of the relationship. Positive and negative actions in the early phase were included in separate models to reduce the number of predictors in each model. Interactions with age were not included because preliminary analyses indicated a high correlation, *r*(26) = .67, *p* = .0001, between age and positive actions in the first phase, which may confound the results. A preliminary analysis found no gender differences for any of the sequences, all *p*s > .12, or positive and negative actions in the first phase, all *p*s > .86, and thus gender was not included in the CCMMs.

Although the child/dyad sample size is small, at 28 children it is close to the 30 units or larger generally recommended for MLM [22, 23]. Furthermore, a power analysis was run using Optimal Design Software. The power of finding a statistically significant small effect is 80% based on the sample in the current study. Finally, this study included over 1000 play sessions and the level of analysis in the present study is the session level nested within individuals within dyads.

## 3. Results

### 3.1 Descriptive statistics

Table 1 provides a correlation matrix for predictor and dependent variables. Age had a positive moderate correlation with conflict frequency. However, no correlation was found between age and games frequency. Age had a strong positive correlation with positive actions in the early phase. There was no correlation between age and negative actions in the early phase. A trend was found for a moderate positive correlation between positive and negative actions in the early phase, *r*(26) = .33, *p* = .08.

**Table 1:** Correlation matrix of independent and dependent variables.

	1	2	3	4
1. Age				
2. Games Freq	.07			
3. Conflicts Freq	.47*	.23		
4. 1^st^ Positive	.67**	.39*	.73**	
5. 1^st^ Negative	.15	-.02	.52**	.33

* *p* < .05

** *p* < .01

### 3.2 Frequency of games

When examining the frequency of games, significant amounts of the variance were explained at the session- and dyad-levels in all models. The AIC estimate was lowest for Model 1 in which 35% of the random variance was at the dyad-level and 65% at the session-level. The proportional reduction in prediction error for the session-level from the Null Model to Model 1 was 3.6%. In Model 1, the dummy coded late phase (*B* = .73, *p* = .0001), but not middle, positively predicted the frequency of games.

**Fig 1:**
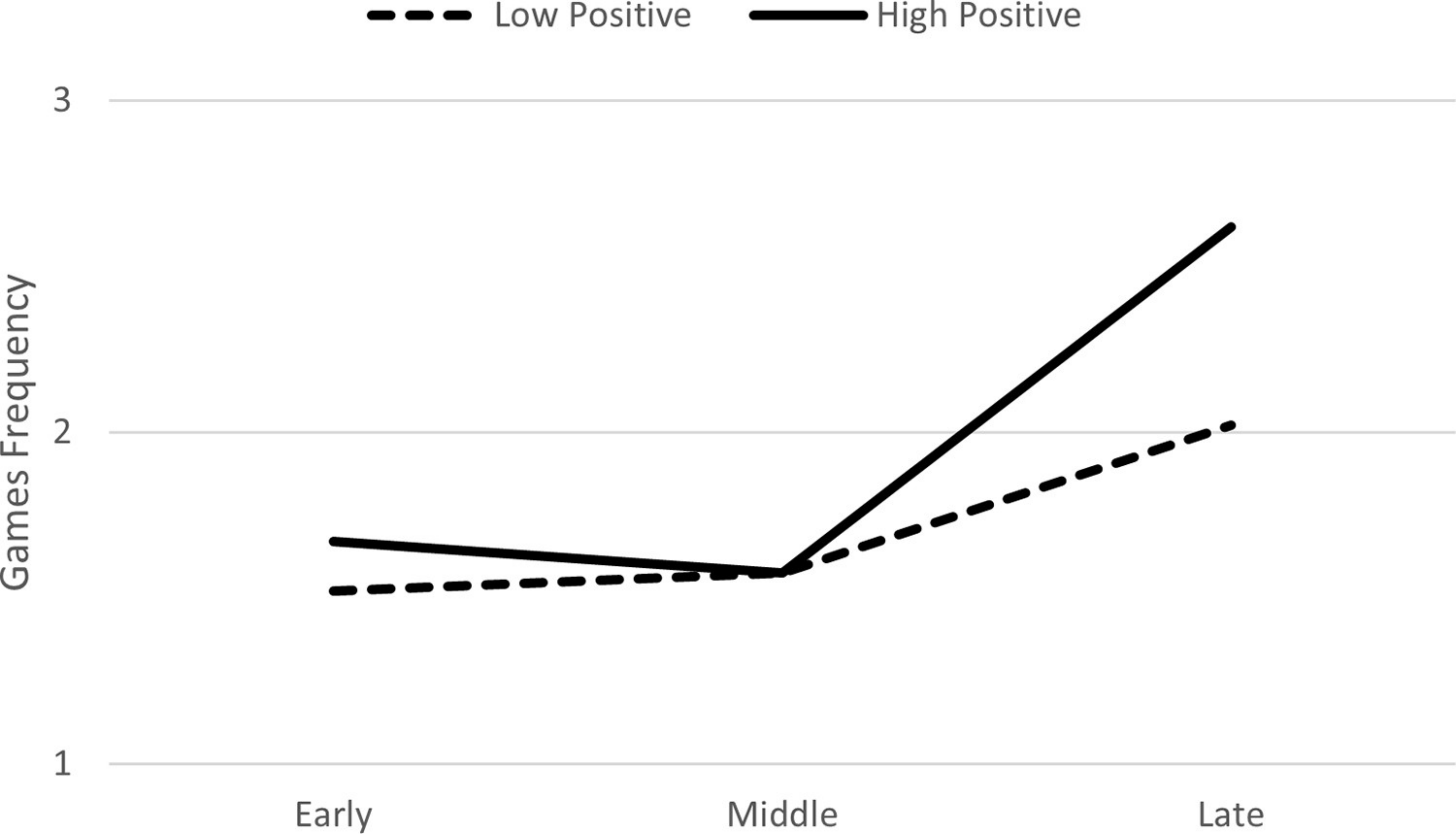
Change over time in frequency of games as function of positive actions in the first phase of the relationship.

In Model 2, when positive actions were entered as a predictor, there was a trend for an interaction between positive actions and the late phase (*B* = .03, *p* = .06), which positively predicted the frequency of games (Table 2). In the follow-up analysis comparing the early and late phases to the middle phase, the interaction between positive actions and the late phase (*B* = .04, *p* = .03), but not the interaction with the early phase, positively predicted the frequency of games. This suggests that frequency of games did not change from the early to middle phase, but significantly increased from middle to late phase. Graphing this interaction based on guidelines by Aiken and West [24], using +/- 1 *SD* for positive actions suggested that the increase in the frequency of games from the middle to late phase was observed particularly for toddlers who had a high frequency of positive actions in the first phase (Fig 1).

**Table 2:** Cross-classified multilevel model examining frequency of game sequences with phase, positive actions in the first phase, and age as predictors.

	Null Model		Model 1		Model 2	
	Est.	SE	Est.	SE	Est.	SE
**Fixed Effects**						
Middle phase			.04	.14	.12	.27
Late phase			.73*	.14	.30	.27
Positive actions in first phase					.01	.02
Age					.01	.04
Positive actions X Middle					-.01	.02
Positive actions X Late					.03^	.02
Intercept	1.68*	.25	1.43*	.26	1.19	1.07
**Random Effects** (variance decomposition)						
Dyad	1.60* (34%)	.47	1.60* (35%)	.47	1.54* (35%)	.47
Child	0.00 (0%)	0.00	0.00 (0%)	0.00	0.00 (0%)	0.00
Session	3.08*(66%)	.14	2.97* (65%)	.14	2.97* (65%)	.14
AIC	3940.78		3913.15**		3938.15	

^*p* = .06

*p < .05

** best model fit based on AIC

**Fig 2:**
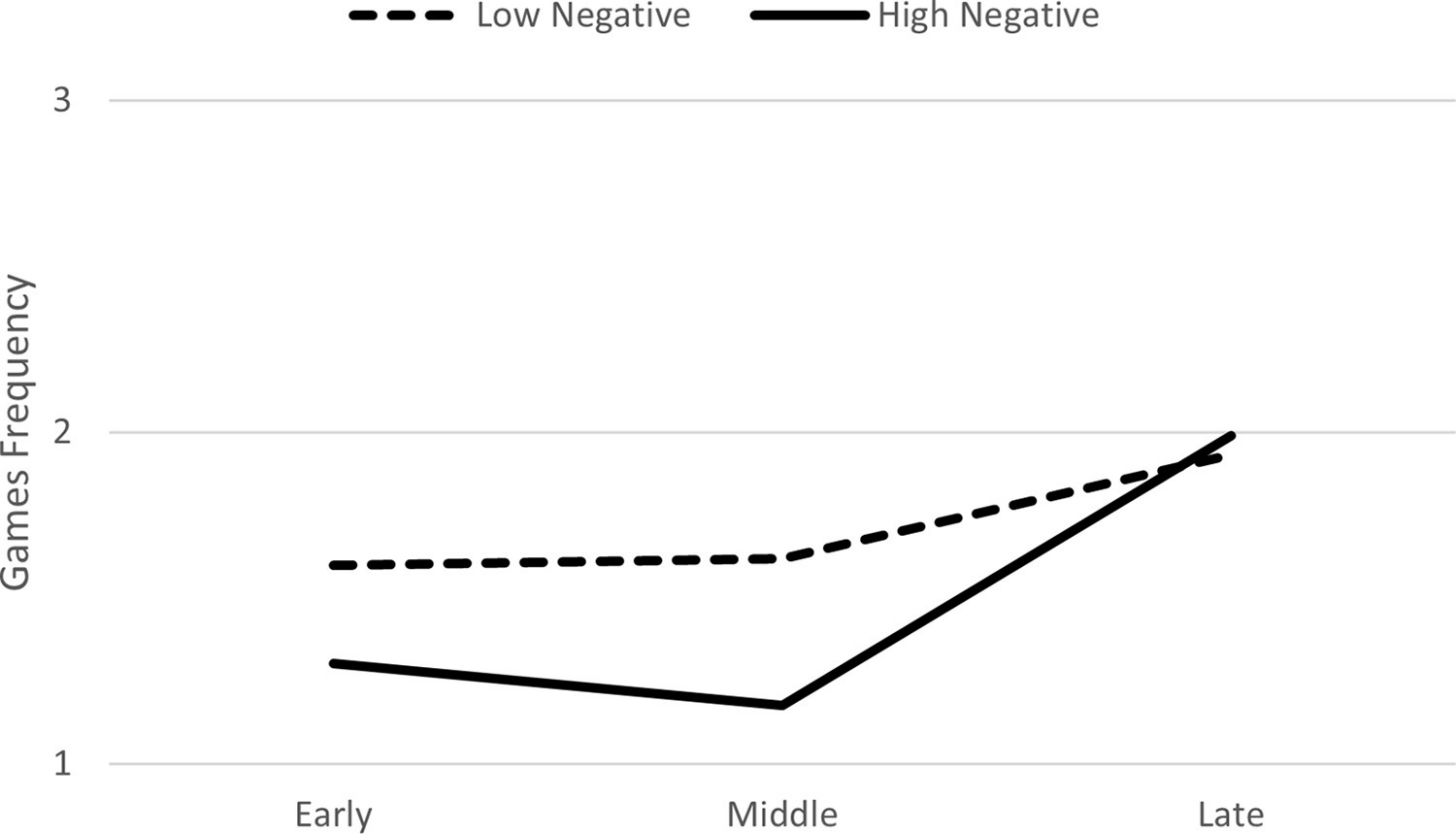
Change over time in frequency of games as function of negative actions in the first phase of the relationship.

In Model 2, when negative actions were entered as a predictor, there was a significant interaction between negative actions and the late phase (*B* = .06, *p* = .0001), which positively predicted the frequency of games (Table 3). In the follow-up analysis comparing the early and late phases to the middle phase, the interaction between negative actions and the late phase (*B* = .06, *p* = .0001), but not the interaction with the early phase, positively predicted the frequency of games. This suggests that frequency of games did not change from the early to middle phase, but significantly increased from middle to late phase. Graphing this interaction based on guidelines by Aiken and West [24], using +/- 1 *SD* for negative actions indicated that the increase in the frequency of games from the middle to late phase was observed particularly for toddlers who had a high frequency of negative actions in the first phase (Fig 2).

**Table 3:** Cross-classified multilevel model examining frequency of game sequences with phase, negative actions in the first phase, and age as predictors.

	Null Model		Model 1		Model 2	
	Est.	SE	Est.	SE	Est.	SE
**Fixed Effects**						
Middle phase			.04	.14	.07	.24
Late phase			.73*	.14	.04	.24
Negative actions in first phase					-.02	.02
Age					.01	.04
Negative actions X Middle					-.01	.02
Negative actions X Late					.06*	.02
Intercept	1.68*	.25	1.43*	.26	1.43	1.10
**Random Effects** (variance decomposition)						
Dyad	1.60* (34%)	.47	1.60* (35%)	.47*	1.65*(36%)	.49
Child	0.00 (0%)	0.00	0.00 (0%)	0.00	0.00 (0%)	0.00
Session	3.08* (66%)	.14	2.97* (65%)	.14	2.92* (64%)	.14
AIC	3940.78		3913.15**		3928.36	

*p < .05

** best model fit based on AIC

### 3.3 Frequency of conflicts

When examining the frequency of conflicts, significant amounts of the variance were explained at the session- and dyad-levels in all models. The AIC estimate was lowest for Model 1, in which 47% of the variance was at the dyad-level and 53% at the session-level. The proportional reduction in prediction error for the session-level from the Null Model to Model 1 was .80%. In Model 1, the dummy coded middle phase (*B* = -.94, *p* = .002), but not late, negatively predicted the frequency of conflicts.

**Fig 3:**
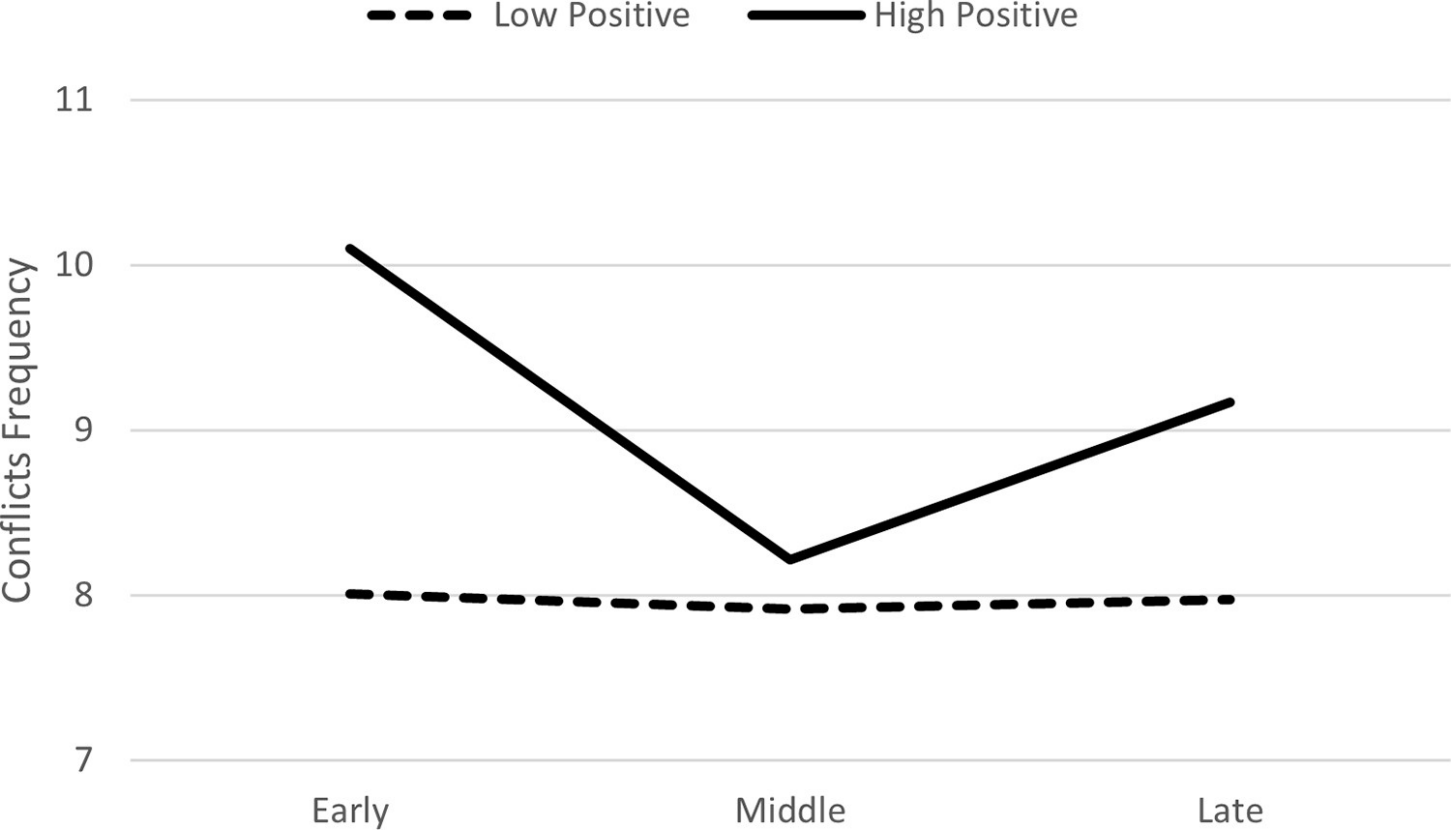
Change over time in frequency of conflicts as function of positive actions in the first phase of the relationship.

In Model 2, when positive actions were entered as a predictor, there was a significant interaction between positive actions and the middle phase (*B* = -.12, *p* = .002), which negatively predicted the frequency of conflicts (Table 4). In the follow-up analysis comparing the early and late phases to the middle phase, the interaction between positive actions and the early phase (*B* = .04, *p* = .03), but not the interaction with the late phase, positively predicted the frequency of conflicts. This indicated that the frequency of conflicts decreased from the early to middle phase and then remained stable. Graphing this interaction based on guidelines by Aiken and West [24], using +/- 1 *SD* for positive actions suggested that the decrease in the frequency of conflicts from the early to middle phase was observed particularly for toddlers who had a high frequency of positive actions in the first phase (Fig 3).

**Table 4:** Cross-classified multilevel model examining frequency of conflict sequences with phase, positive actions in the first phase, and age as predictors.

	Null Model		Model 1		Model 2	
	Est.	SE	Est.	SE	Est.	SE
**Fixed Effects**						
Middle phase			-.94*	.30	.70	.60
Late phase			-.43	.30	.36	.60
Positive actions in first phase					.14*	.05
Age					.16	.09
Positive actions X Middle					-.12*	.04
Positive actions X Late					-.06	.04
Intercept	8.45*	.71	8.90*	.74	2.82	2.60
**Random Effects** (variance decomposition)						
Dyad	13.36* (47%)	3.82	13.37* (47%)	3.82	9.99* (40%)	3.05
Child	0.00 (0%)	0.00	0.00 (0%)	0.00	0.00 (0%)	0.00
Session	15.16* (53%)	.70	15.04* (53%)	.69	14.97* (60%)	.69
AIC	5500.89		5496.78**		5506.27	

*p < .05

** best model fit based on AIC

**Fig 4:**
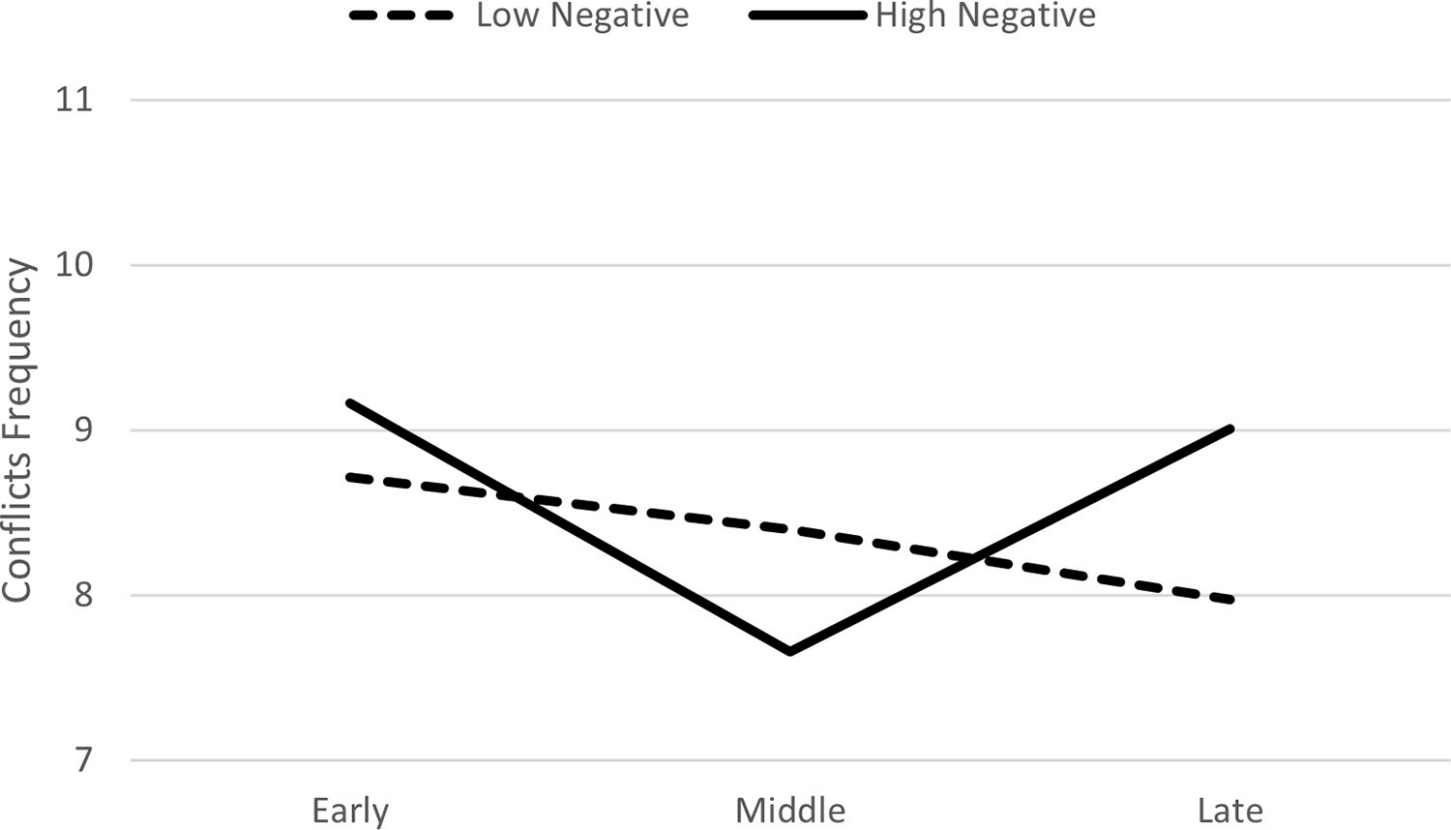
Change over time in frequency of conflicts as function of negative actions in the first phase of the relationship.

In Model 2, when negative actions were entered as a predictor, there was a significant interaction between negative actions and the middle phase (*B* = -.08, *p* = .02), which negatively predicted the frequency of conflicts (Table 5). In the follow-up analysis comparing the early and late phases to the middle phase, the interaction between negative actions and the late phase (*B* = .09, *p* = .008), but not the interaction with the early phase, positively predicted the frequency of conflicts. This shows that frequency of conflicts decreased from the early to middle phase and then increased from the middle to late phase. Graphing this interaction based on guidelines by Aiken and West [24], using +/- 1 *SD* for negative actions suggested that the decrease in the frequency of conflicts from the early to middle phase and then increase again in the late phase was observed particularly for toddlers who had a high frequency of negative actions in the first phase (Fig 4).

**Table 5:** Cross-classified multilevel model examining frequency of conflict sequences with phase, negative actions in the first phase, and age as predictors.

	Null Model		Model 1		Model 2	
	Est.	SE	Est.	SE	Est.	SE
**Fixed Effects**						
Middle phase			-.94*	.30	.07	.54
Late phase			-.43	.30	-.94	.54
Negative actions in first phase					.03	.04
Age					.14	.10
Negative actions X Middle					-.08*	.04
Negative actions X Late					.04	.04
Intercept	8.45*	.71	8.90*	.74	4.84	2.73
**Random Effects** (variance decomposition)						
Dyad	13.36* (47%)	3.82	13.37* (47%)	3.82	11.55*(44%)	3.52
Child	0.00 (0%)	0.00	0.00 (0%)	0.00	0.00 (0%)	0.00
Session	15.16* (53%)	.70	15.04* (53%)	.69	14.92* (56%)	.69
AIC	5500.89		5496.78**		5507.71	

*p < .05

** best model fit based on AIC

## 4. Discussion

The present study explored the formation of relationships among toddlers. Specifically, we examined whether behaviors early in the relationship predicted interactions later in the relationship. The main findings indicated that both positive and negative behaviors at the beginning of the relationship predicted a higher frequency of games sequences in the later part of the relationship. With respect to conflict sequences, positive behaviors at the beginning of the relationship predicted fewer conflicts in the middle phase and then remained stable. Negative behaviors at the beginning predicted more conflicts later in the relationship. These findings provide an important contribution to research on the development of very young children’s friendships, which are important for later social, emotional, and cognitive functioning [2]. These very early close, reciprocal relationships are important for adult mental and physical health [4].

While there were no correlations with age and the frequency of negative actions in the early phase, there was a strong positive correlation between age and frequency of positive actions in the early phase. It is possible that older toddlers have been socialized with respect to positive behaviors that are expected in interactions with peers. Yet, negative actions might be more difficult to regulate [25, 26] even among older toddlers.

The frequency of games later in the relationship was higher not only for toddlers with more positive actions in the first phase, but also for toddlers with more negative actions in the first phase. Although the latter finding was not consistent with our hypothesis, it is in line with previous research on friendships and their multiplex nature. Indeed, Ross and Conant [10] found that toddlers who tended to engage in disputes with other toddlers also had more positive interactions with them. Furthermore, preschool aged children engage in more conflicts with their friends than non-friends [8, 9]. Hay et al. [27] recently examined the parallel development of prosocial behavior and aggression among unfamiliar toddlers. The results indicated that these positive and negative actions were positively correlated, a finding that suggests a general sociability factor for these behaviors [27].

When comparing Figs 1 and 2, it appears that the frequency of games in the late phase of the relationship, was slightly higher for toddlers who had high positive actions in the first phase compared to those with high negative actions. Thus, high frequency of positive interactions in the beginning of the relationship is particularly important for later positive and enjoyable interactions (i.e., games).

Our hypotheses regarding conflict sequences were confirmed. As predicted, while initial negative actions predicted increased frequency of conflicts later in the relationship, initial positive actions predicted fewer conflicts as toddlers became acquainted. These findings are consistent with previous research suggesting that initial negative interactions reduce the likelihood of forming cooperative relationships [5–7]. Our study extends this body of work to the toddler developmental period. Unlike previous research, the 18 sessions in our study allowed to examine how early interactions contribute to toddlers’ evolving relationship.

As illustrated by the figures, the frequency of conflicts at the late phase of the relationship, was greater than the frequency of games. This finding was observed particularly among more “interactive” toddlers (i.e., those that tended to be high on both positive and negative actions).

Some potential limitations of the present study should be noted. First, given the archival nature of the data set, the findings may be subject to cohort effects. Since the present study focused on basic interactions among young toddlers, the likelihood of such a cohort effect may be less of a concern than if the focus was on more contextual factors. Second, the parents in our sample were relatively well-educated and none of the children were attending any formal childcare. Thus, the study results may not generalize to toddlers with less educated parents and those who form relationships within a childcare setting. However, most Canadian 1–3-year-olds do not attend formal daycare centers [28] and the majority of Canadians complete high school [29]. Furthermore, focusing on a sample of children who were cared for at home increased the homogeneity of our sample in terms of their social experiences possibly increasing the likelihood that we could identify trends in interactions as children got to know one another without substantially increasing our sample size. Third, we examined total positive and negative in the first phase of the relationship. Future research should investigate specific types of positive and negative actions, as well as initiations versus other contributions (e.g., responses to initiations). Finally, the number of participants in the present study was small. Nevertheless, the study included over 1000 sessions (over 600 hours of observations in total). This intensive longitudinal assessment of each dyad was sufficiently large to produce a very rich and unique set of observations.

In summary, our study shows that both initial positive and negative behaviors contribute to increased frequency of enjoyable activities later in toddler relationships. Furthermore, initial negative behaviors increase the frequency of quarrels later in the relationship; initial positive behaviors reduce their frequency. Taken together, these findings suggest that positive and negative behaviors when toddlers initially meet underlie the pathway in which their relationship develops. Thus, parents and practitioners who work with families should pay particular attention to scaffolding the quality of these early interactions. To the best of our knowledge, this is the first study to examine how initial interactions play a role in their evolving friendship.

## Supporting information

S1 Dataset (XLSX)

S1 Table Effect of quad on frequency of games. (DOCX)

S2 Table Effect of quad on frequency of conflicts. (DOCX)

S1 Fig Average frequency of games over time for each participant. (TIF)

S2 Fig Average frequency of conflicts over time for each participant. (TIF)
