# Supplementary material for: Positive and negative actions early in the relationship predict later interactions among toddlers
Source: PLoS One. 2022 Nov 3;17(11):e0276932. doi: 10.1371/journal.pone.0276932 (PMC9632877; doi:10.1371/journal.pone.0276932)
Supplement: S1 Table — (DOCX) [file pone.0276932.s002.docx]

Supplementary Table 1

Effect of quad on frequency of games

| **Parameters** | Model 1 | Quad 1 20 months male | Quad 1 30 months female | Quad 2 20 months female | Quad 2 30 months male | Quad 3 30 months female | Quad 4 20 months male | Quad 4 30 months male |
| --- | --- | --- | --- | --- | --- | --- | --- | --- |
| **Fixed Effects** |  |  |  |  |  |  |  |  |
| Middle phase | .04 | .04 | .04 | .04 | .04 | .04 | .04 | .04 |
| Late phase | .73* | .73* | .73* | .73* | .73* | .73* | .73* | .73* |
| Quad |  | .09 | -.62 | .24 | 1.61* | -.61 | -.54 | -.21 |
| Intercept | 1.43* | 1.41* | 1.52* | 1.39* | 1.19* | 1.51* | 1.51* | 1.45* |
| **Random Effects** |  |  |  |  |  |  |  |  |
| Dyad | 1.60* | 1.67* | 1.62* | 1.66* | 1.31* | 1.62* | 1.63* | 1.66* |
| Child | 0.00 | 0.00 | 0.00 | 0.00 | 0.00 | 0.00 | 0.00 | 0.00 |
| Session | 2.97* | 2.97* | 2.97* | 2.97* | 2.97* | 2.97* | 2.97* | 2.97* |

**p* < .05
