# Supplementary material for: Positive and negative actions early in the relationship predict later interactions among toddlers
Source: PLoS One. 2022 Nov 3;17(11):e0276932. doi: 10.1371/journal.pone.0276932 (PMC9632877; doi:10.1371/journal.pone.0276932)
Supplement: S2 Table — (DOCX) [file pone.0276932.s003.docx]

Supplementary Table 2

Effect of quad on frequency of conflicts

| **Parameters** | Model 1 | Quad 1 20 months male | Quad 1 30 months female | Quad 2 20 months female | Quad 2 30 months male | Quad 3 30 months female | Quad 4 20 months male | Quad 4 30 months male |
| --- | --- | --- | --- | --- | --- | --- | --- | --- |
| **Fixed Effects** |  |  |  |  |  |  |  |  |
| Middle phase | -.94* | -.94* | -.94* | -.94* | -.94* | -.94* | -.94* | -.94* |
| Late phase | -.43 | -.43 | -.43 | -.43 | -.43 | -.43 | -.43 | -.43 |
| Quad |  | -3.19 | -2.92 | -4.43* | 1.91 | 3.76* | 2.05 | 3.60 |
| Intercept | 8.90* | 9.37* | 9.33* | 9.56* | 8.62* | 8.34* | 8.60* | 8.50* |
| **Random Effects** |  |  |  |  |  |  |  |  |
| Dyad | 13.37* | 12.53* | 12.75* | 11.25* | 13.42* | 12.01* | 13.34* | 12.53* |
| Child | 0.00 | 0.00 | 0.00 | 0.00 | 0.00 | 0.00 | 0.00 | 0.00 |
| Session | 15.04* | 15.04* | 15.04* | 15.04* | 15.04* | 15.04* | 15.04* | 15.04* |

**p* < .05
