# Supplementary figures and images for: Positive and negative actions early in the relationship predict later interactions among toddlers
Source: PLoS One. 2022 Nov 3;17(11):e0276932. doi: 10.1371/journal.pone.0276932 (PMC9632877; doi:10.1371/journal.pone.0276932)

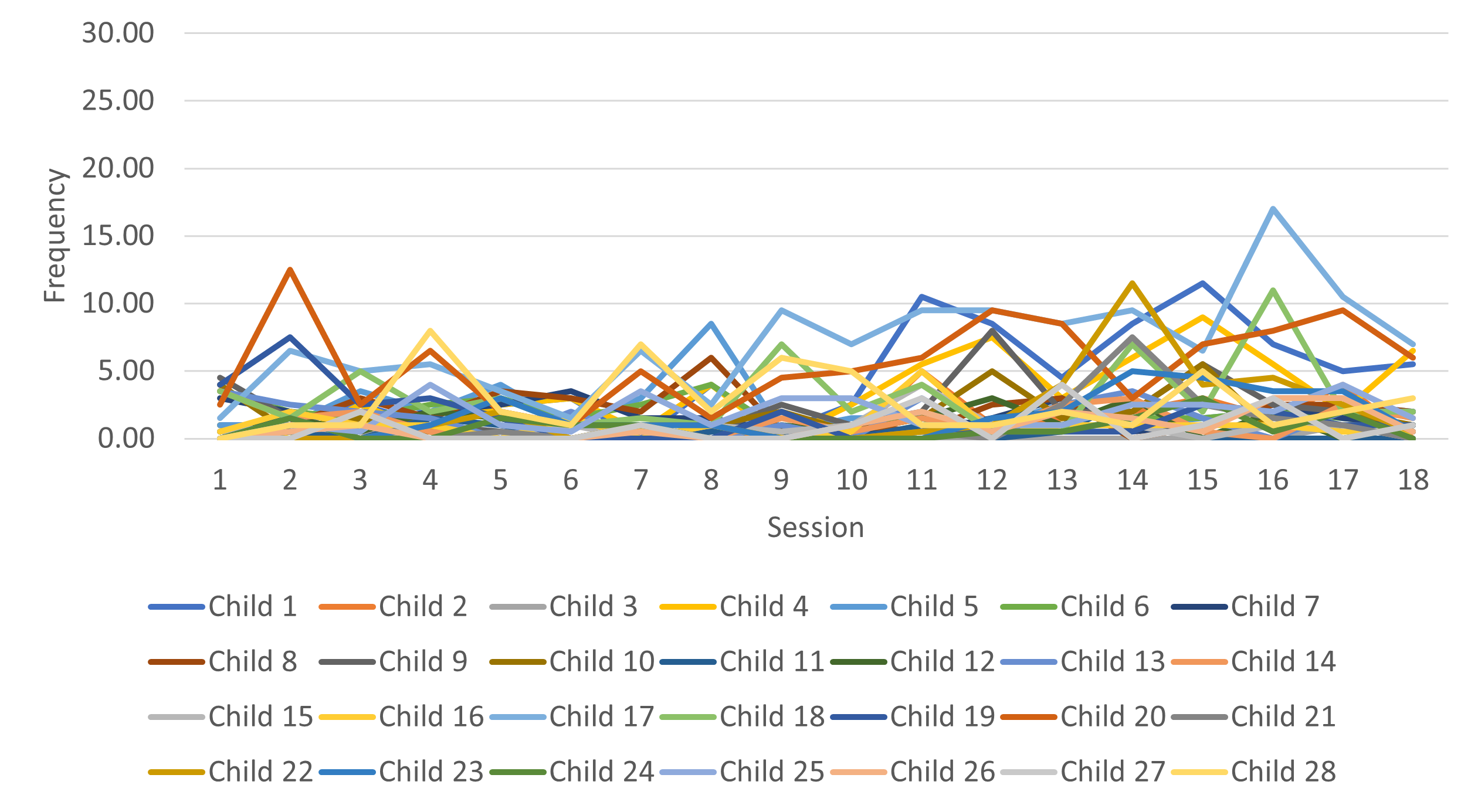

Supplement: S1 Fig — (TIF) [file pone.0276932.s004.tif]

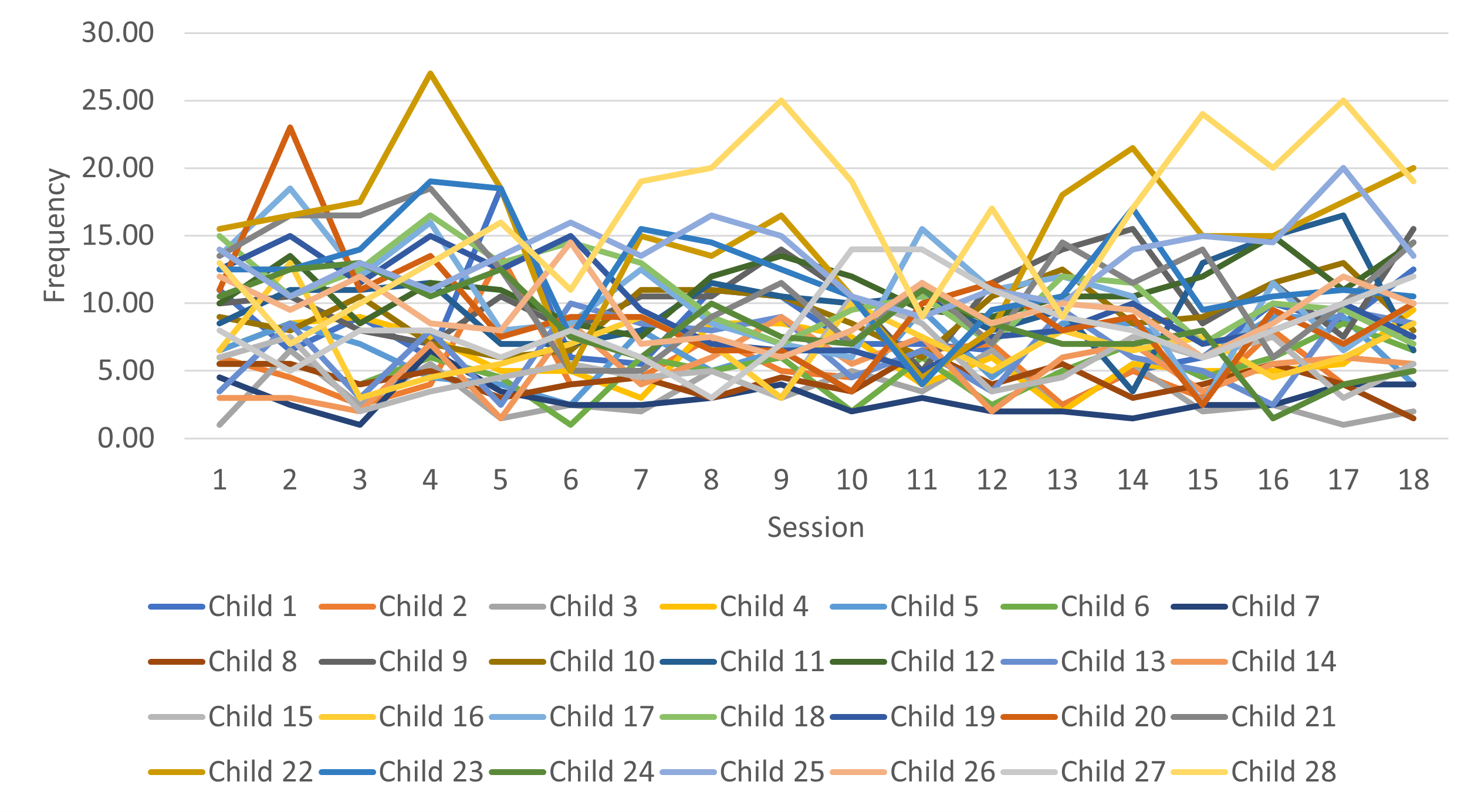

Supplement: S2 Fig — (TIF) [file pone.0276932.s005.tif]
